# An SVM-Based NAND Flash Endurance Prediction Method

**DOI:** 10.3390/mi12070746

**Published:** 2021-06-25

**Authors:** Haichun Zhang, Jie Wang, Zhuo Chen, Yuqian Pan, Zhaojun Lu, Zhenglin Liu

**Affiliations:** 1 School of Optical and Electronic Information, Huazhong University of Science and Technology, Wuhan 430074, China; D201880640@hust.edu.cn (H.Z.); zoarzzz@outlook.com (Z.C.); 2 Shenzhen Kaiyuan Internet Security Technology Co., Ltd., Shenzhen 518000, China; wangjie@seczone.cn; 3 School of Cyber Science and Engineering, Huazhong University of Science and Technology, Wuhan 430074, China; yuqianpan@126.com (Y.P.); lzj_cse@hust.edu.cn (Z.L.)

**Keywords:** NAND flash memory, test platform, endurance, support vector machine, raw bit error

## Abstract

NAND flash memory is widely used in communications, commercial servers, and cloud storage devices with a series of advantages such as high density, low cost, high speed, anti-magnetic, and anti-vibration. However, the reliability is increasingly getting worse while process improvements and technological advancements have brought higher storage densities to NAND flash memory. The degradation of reliability not only reduces the lifetime of the NAND flash memory but also causes the devices to be replaced prematurely based on the nominal value far below the minimum actual value, resulting in a great waste of lifetime. Using machine learning algorithms to accurately predict endurance levels can optimize wear-leveling strategies and warn bad memory blocks, which is of great significance for effectively extending the lifetime of NAND flash memory devices and avoiding serious losses caused by sudden failures. In this work, a multi-class endurance prediction scheme based on the SVM algorithm is proposed, which can predict the remaining P-E cycle level and the raw bit error level after various P-E cycles. Feature analysis based on endurance data is used to determine the basic elements of the model. Based on the error features, we present a variety of targeted optimization strategies, such as extracting the numerical features closely related to the endurance, and reducing the noise interference of transient faults through short-term repeated operations. Besides a high-parallel flash test platform supporting multiple protocols, a feature preprocessing module is constructed based on the ZYNQ-7030 chip. The pipelined module of SVM decision model can complete a single prediction within 37 us.

## 1. Introduction

With the development of smart devices and cloud computing, flash memory has gained great popularity in various fields [1]. NAND flash memory has achieved larger storage capacity and higher storage speed than NOR flash memory by virtue of the design mode of storage units connected in series, becoming an important large-scale data storage medium. In order to pursue higher storage density, a variety of technologies have been developed in the field of NAND flash memory. Three-dimensional structure technology [2] is committed to transforming a planar structure into a three-dimensional structure, which increases the storage capacity under the same area. Multi-bit memory cell technology [3] focuses on improving the number of bits in the storage unit in order to achieve a multiple increase in storage capacity. With gradual in-depth study of the two technologies, researchers have found that while the storage density of NAND flash memory has doubled, the data reliability problem has worsened.

Data reliability marks the accuracy of data storage. If data errors occur during use, serious consequences will be immeasurable. In the field of NAND flash memory, data reliability problems are mainly reflected in retention [4] and endurance. The former reflects the data retention time without re-erasing, while the latter is the problem of reliability degradation caused by structural damage to the memory cell during use. Compared to retention, endurance has a greater impact on the actual product. As the number of programming increases, the endurance of the flash memory decreases and the number Raw Bit Error (RBE) gradually rises. The RBE number is the number of bits of difference between the actual read data and the actual programmed data without error correction, which is an important parameter to characterize the degree of endurance change. When the RBE numbers exceeds a certain limit, the flash memory will not continue to be used normally.

It has become an important direction for academia to suppress data errors caused by reduced endurance. The Error Correcting Code (ECC) error correction algorithm uses special coding rules to check and correct the original data [5], so the endurance of the flash memory can be indirectly improved by optimizing the error correction algorithm. However, limited by the storage space, the optimization effect is not ideal. Wear-Leveling technology [6] indirectly delays the occurrence of user data failure by balancing the number of programming and erasing of each block. However, the total endurance of the regions in the flash memory is not equal; there is room for further optimization of the technology. The Read-Retry technology reduces data errors by modifying the hard-decision reference voltage of the read operation, but this will increase the operating time and reduce the performance.

However, the real dilemma of flash memory reliability research lies in the uncertainty of endurance, which will lead to huge waste. The minimum actual endurance of flash memory is often dozens or even a hundred times the manufacturer’s nominal value [7]. The reason why manufacturers specify the nominal value so conservatively is the huge difference in endurance between the same type of flash memory particles [8]. Even if the manufacturer inspects and screens the wafers before they leave the factory, there are still several times or even ten times the endurance difference between the same model and the same batch of flash memory particles. Besides, the particle-level inspection is destructive and extremely time-consuming. If the endurance can be accurately predicted and an early warning be made, the user can adjust the critical value of the data transfer process and greatly extend the service life of the flash memory.

Using machine learning algorithms to accurately predict changes in flash memory endurance-related parameters have become an important means to solve the flash memory reliability dilemma. It can greatly optimize the existing flash memory management strategies and implement accurate endurance warnings. Damien Hogan tried to combine a supervised Genetic Programming (GP) algorithm with the endurance prediction of 2D Multi-level Cell (MLC) flash memory to determine whether the sample flash memory with different levels of Program-Erase (P-E) cycles will generate uncorrectable data errors [9]. However, the GP two-category prediction model finally obtained in the study has a prediction accuracy of only 83.5% on the test set when the decision boundary is 35,000. Barry Fitzgerald observed through a large amount of experimental data that the word line (WL) number, page type, and page parity in MLC flash memory will affect the code word error rate (CWER), the programming, and erasing duration [10]. Using the feature, the study proposed a sampling method based on the error probability density function [11], and constructed eight different two-class machine learning models. However, the study neglected the class balance of the data set. The number of negative samples representing the number of codeword errors exceeding 100 only accounted for 0.03% of the total number of samples, which led to a significant decrease in the reliability of the model accuracy results. Ruixiang Ma considered that the predictive model may lose its validity due to changes in the flash memory usage environment, so the incremental changes in endurance parameters are used to update the predictive model to adapt to the parameter changes at different endurance stages [12]. However, this solution did not take into account the hardware complexity and application limitations of using the same flash memory pre-data to predict the later endurance.

On the one hand, the endurance prediction model established by existing studies performs two-class prediction of the RBE numbers, and the RBE numbers corresponding to the classification boundary is close to the upper limit of the ECC error correction algorithm, which limits the application scenarios of the prediction model. On the other hand, existing research does not consider the disturbance of electrical effects such as transient errors in NAND flash memory on the prediction results, which greatly reduces the prediction accuracy. In order to solve the endurance prediction problem, this paper designs a set of NAND flash memory endurance class prediction method based on SVM algorithm based on a large amount of experimental test data and combined with micro-mechanism analysis. The main contents of the paper are as follows:

(1) The multi-classification model describes the endurance level with the remaining P-E cycle number and RBE numbers. The output result has multiple levels and labels, which greatly expands the application scenarios.
(2) This paper has carried out a number of targeted optimization strategies for the disturbance factors in the flash memory. By preprocessing the input features, the prediction model still has a high accuracy rate when the output categories increase.
(3) We further use FPGA to implement the SVM prediction circuit module, which can be actually applied to solid-state hard disk controller chips. Compared with common embedded software implementations, the prediction circuit module designed in the paper has a faster prediction speed, with less hardware resources, and has broad application prospects.

## 2. Flash Error Mechanism and Prediction Algorithm

### 2.1. Flash Error Mechanism

The cause of the NAND flash memory endurance problem lies in its unique memory cell and array structure. The basic memory cell structure of flash memory is based on the development and evolution of the Floating Gate (FG) Field Effect Transistor (FET). The internal electrical disturbance phenomenon of the NAND flash memory is closely related to its array structure. The NAND flash memory will produce the decrease of reliability due to many physical effects in actual scenarios. There are several kinds of mechanisms.

#### 2.1.1. Unit Wear-Out

Unit cell wear is the direct cause of reduced endurance of flash memory. Wear-out and aging often occur in the process of charge tunneling and transfer during programming and erasing operations. Wear-out causes the atomic bonds at the interface between the charge trap layer and the insulating layer to break, resulting in interface traps that interfere with the charge transport process and cause the threshold voltage to deviate from the ideal value. Therefore, the interface traps are the main reason for the endurance of the flash memory cell to decrease and the occurrence of error bit flips. The wear-out and aging of the unit caused by the P-E cycle is small but irreversible, and the number of P-E cycles is correlated with the endurance.

#### 2.1.2. Disturbance

Certain electrical effects caused by the special array structure of flash memory can cause threshold voltage shifts. The most common effect is the disturbance phenomenon [13]. Disturbance is not permanently structurally destructive. The memory cell is restored to its original state by erasing. And the severity of disturbance is closely related to the programming pattern. The specific programming pattern significantly stimulates some disturbances. Multiple read operations on the same memory cell before the erase operation will cause reads disturbance, which causes the threshold voltage of the affected memory cell to shift in the positive direction. When the shifted threshold voltage exceeds the hard-decision reference voltage between different programming states, data errors will occur. Program disturbance and pass disturbance will occur during the programming operation. Edge word line disturbance also occurs during programming operations. The edge word line unit generates a large number of electron-hole pairs due to the large gate-induced drain leakage [14]. The electrons are accelerated to the channel and injected into the storage layer in the edge word line unit, leading to a surge in threshold voltage and data errors.

#### 2.1.3. Transient Error

Transient error refers to the data error flipping caused by some uncertain transient factors during the operation of the flash memory. Because of the uncertainty of inducing factors, these transient errors are difficult to limit by conventional means. The most typical transient error is the uncertainty error caused by the Random Telegraph Noise (RTN) phenomenon. The error causes uncertain fluctuations in the drain current [15], which in turn causes the threshold voltage to fluctuate uncertainly.

### 2.2. Prediction Algorithm

The SVM algorithm has been developed rapidly through the efforts of many scholars since it was proposed in 1963. It is based on slack variables [16] and VC dimension [17], and has strong sparsity and generalization capabilities. The ultimate goal of linear SVM is to find the optimal hyperplane $P_{k}:\omega^{T}X+b=0$ that can divide the sample space correctly. However, the actual classification problem is often a non-linear problem, which requires a certain non-linear transformation to achieve spatial upscaling, so that a hyperplane that can be correctly classified in the high-dimensional feature space reappears. The kernel function can reduce the complexity of high-dimensional inner product operations. The SVM algorithm often uses Radial Basis Function as a kernel function in practical applications.

The SVM decision model only supports two classifications. The One-against-One (OAO) method [18] groups the training data according to the output categories, and builds a separate two-class SVM model between each two categories. A total of K(K−1)/2 decision boundaries are obtained, and K is the total number of categories. When facing the new data to be classified, the OAO method inputs it into K(K−1)/2 models to obtain the corresponding classification results, respectively. Finally, according to the voting strategy, the category with the most votes among all the results is counted as the final classification results.

The Decision Tree (DT) classifier can perform fast and effective classification in the face of a large amount of data input. However, weak generalization will cause serious overfitting in the sample space where the total number of categories is unbalanced. At the same time, the instability of the DT classifier will cause it to be very sensitive to high-frequency jitter in the flash memory training data, and generate very different DT models.

The implementation method, the K-Nearest Neighbor (KNN) classifier, is simple, and for the simplest KNN classifier, it does not even need to be trained. But if the training set sample points are not clipped, the method needs to store all sample points and calculate the distance from the sample points to be classified to all the sample points. The storage space and calculation resources required are very huge, which is not suitable for circuit realization.

## 3. Endurance Prediction Method

### 3.1. The Process of Endurance Prediction Method

**Figure 1:**
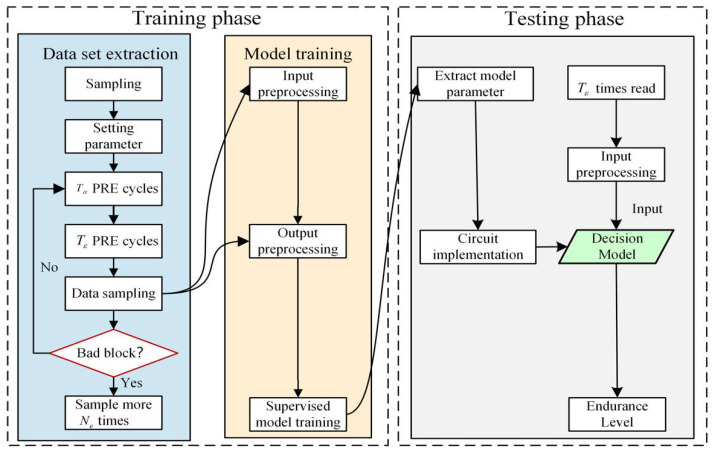
The process of SVM-based endurance prediction method.

Figure 1 shows the process of the SVM-based endurance prediction method; the SVM-based NAND Flash endurance prediction method includes two phases: Training phase and testing phase. The purpose of the training phase is to obtain sample data sets and use machine learning algorithms to establish a decision model suitable for Flash endurance prediction. The testing phase uses the established decision model to predict endurance.

#### 3.1.1. Training Phase

Training phase includes data set extraction and model training. Data set extraction performs repeated P-E cycles on memory blocks of different flash memory particles with the same model to obtain flash memory endurance-related data in a certain rule. Model training uses the acquired flash memory endurance-related data set to train the machine learning model to obtain the decision function.

(a) Sample Selection

Select a certain number of flash memory blocks of the same model with suitable locations as samples. In order to avoid over-fitting, the number of flash memory blocks selected in each flash memory particle needs to be consistent.

(b) Parameter Setting

Set flash memory specification information, including interface protocol type, storage unit type, block size, page size, and the total number of blocks in a single logical unit. At the same time, set the test information, such as the used programming pattern, test mode, bad block characteristic error rate, etc. The random programming pattern can simulate various programming levels and combinations, fitting the programming pressure in practical applications to the greatest extent, which sets it as the default programming pattern in the proposed prediction method.

(c) P-E Cycle

$T_{\alpha }$ P-E cycles are performed on flash memory particles to be tested to accelerate the wear of the memory unit. The number of P-E cycles $N_{c}$ will be increased by one each time a cycle is completed. When performing P-E cycles on flash memory particles to accelerate memory cell wear, it is necessary to keep the idle period interval between programming and erasing operations in each cycle fixed to eliminate the difference in actual endurance caused by different idle period intervals.

(d) P-R-E Cycle and Data Sampling

$T_{\varepsilon }$ Program-Read-Erase (P-R-E) cycles are performed on the flash memory particles to be tested to obtain the data required for modeling. Update the current cycle number $N_{c}=N_{c}^{\prime }+T_{\varepsilon }$, and $N_{c}^{\prime }$ is the cycle number before the update. Multiple P-R-E cycle operations in a short period of time help reduce the negative effects of transient errors. Each P-R-E cycle compares the read data with the written original data to obtain the RBE numbers of each flash page. At the same time, the current cycle number $N_{c}$ and the duration of the flash operation are recorded during each P-R-E cycle as the original model training data set.

Repeat steps c and d until it is detected that the RBE numbers of a page in the block exceeds the ECC error correction. In order to ensure that there are enough samples in each endurance stage, continue to perform $T_{e}$ PE cycles and then stop sampling.

#### 3.1.2. Testing Phase

The main purpose of the actual application process of the model is to extract the parameters of the trained model and implement it with a specific circuit, and then face the new data in the actual use scene, call the prediction model circuit, and get the prediction result.

### 3.2. Analysis of Prediction Model

#### 3.2.1. Prediction Object Selection

The object of programming operation is flash memory page, and scholars have focused more on prediction research in the past. However, various disturbances seriously affect the accuracy of these prediction models in actual scenarios. This paper takes the flash memory block as an independent prediction object. The decision was based on the following reasons:

1. Obvious Endurance Difference between Flash Memory Pages

**Figure 2:**
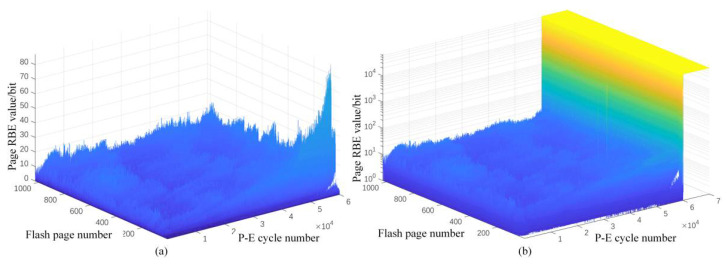
The relationship between the RBE on the page and the P-E cycle number: (**a**) Early and mid in lifecycle; (**b**) End of lifecycle.

As shown in Figure 2a, take Intel’s 29F32B2ALCMG3 NAND Flash particles as an example, different pages show different RBE numbers. In the interval where the page number is lower than 200, the RBE numbers of some pages is significantly higher, showing a more obvious trend of rising with the increase in number of P-E cycles. While other flash memory pages have significantly lower RBE numbers, the change trend is also very irregular. Even after ignoring the high-frequency jitter, there is a local feature where the RBE numbers decreases with the increase in the number of P-E cycles.

The memory cells of different pages have differences in structural size and attributes [19], which leads to inconsistency of the actual tunneling charges suffered by different pages under the same macro programming pressure, directly affecting the degree of cell wear. Moreover, the new three-dimensional multilayer process will bring more serious physical structure differences and disturbance effects [20], resulting in more significant differences in the endurance of flash memory pages. If the flash memory page is used as the prediction object for modeling, the difference in the degree of wear and change trend will greatly reduce the accuracy of the model’s prediction results.

2. The Integrity of the Flash Memory Block

There is also a significant integrity between different pages in the same flash memory block, such as the “cliff” phenomenon. As shown in Figure 2b, even if the wear degree and RBE numbers of different pages are different, the RBE numbers of all pages in the flash memory block jumps at the same time at the end of the life and surges to more than 60,000. Considering that the page capacity of the selected flash is 16 KB and the pattern used is pseudo-random, page RBE numbers as high as half of the page capacity means that the block has lost its storage function. The structural traps generated by the cell wear form fine “cracks” [21], which is very common in many types of flash memory particles. When the “cracks” accumulate to a certain extent, the insulating layer is broken down, forming a penetration path, which causes a large area of memory cells to fail. Pages with large differences in physical characteristics show the same end-of-life endurance performance, which makes it difficult to predict the endurance of the flash memory page as an independent object.

3. Array Coupling and Bad Block Management

On the one hand, the word line wear in a multi-bit memory cell will be reflected in the RBE number of multiple pages, which means that coupling relationship between different pages affects each other. On the other hand, the difference and randomness of the written original data will lead to the difference in the degree of influence of the array interference phenomenon on different pages. The difference is very significant locally, which seriously affects the accuracy of the prediction.

#### 3.2.2. Input Features

The selection and processing of input features determine whether the prediction algorithm can achieve good results in practical applications.

1. Number of P-E Cycles

From the perspective of application scenarios, the number of remaining P-E cycles is a direct indicator of the endurance of the flash memory. Therefore, the number of P-E cycles is the most direct feature of endurance level prediction.

The total endurance of the actual flash memory block is affected by the programming pressure. Factors such as temperature, programming pattern, and idle period time interval will cause differences in programming pressure, resulting in differences in the total endurance of the flash memory block. Process variation can also cause a huge difference in endurance between flash memory particles. Even with the same batch of flash memory particles of the same process, it is impossible to guarantee that all the particles have the same constitution, which results in a large degree of dispersion of the total P-E cycle number between flash memory particles. Thus, the P-E cycle number cannot be used as a single feature for endurance prediction.

2. Raw Bit Errors

RBE measures the degree of unit wear from the perspective of bad block judgment standards. With the increase in the number of P-E cycles, the RBE numbers of each page of the flash memory particle has increased to varying degrees. The dominant reason for the change of RBE numbers is that the interface traps caused by cell wear cause charge escape/combination and cause the threshold voltage to shift.

3. Erasing Duration

Both programming and erasing operations involve the charge tunneling effect. The interface traps caused by the effect will change the electrical parameters of the memory cell, affecting the tunneling efficiency, which indirectly leads to changes in the operating time. The flash programming strategy causes the programming duration to change with the decrease in endurance, but the programming duration as an input feature is not ideal in actual application scenarios due to great differences in different types of pages in the multi-bit memory cell structure.

The erase operation applies a positive pulse on the substrate to initialize all data in the block to an erased state, and also uses threshold voltage verification to determine whether to apply an additional pulse. However, contrary to the programming operation, the interface traps hinder the tunneling of the charge from the storage layer to the substrate and cause the number of erase pulses to increase, which in turn increases the erase duration.

#### 3.2.3. Label

The endurance judgment is related to the ECC error correction algorithm. When the RBE numbers exceeds the upper limit of the algorithm error correction, the endurance will return to zero. In addition, the garbage collection and out-of-place update mechanisms in the SSD controller [22] lead to amplification effects. Therefore, the number of P-E cycles available at the SSD level is much less than the number of P-E cycles available at the flash block level.

According to the endurance criterion described above, the number of P-E cycles is the metric, and the RBE numbers is the criterion. The endurance level prediction model provides a basis for the endurance level evaluation of the SSD wear leveling algorithm. In addition, the endurance level prediction model can be used to warn and mark the flash memory blocks that will become bad blocks. The number of remaining P-E cycles and RBE numbers are both competent for endurance level prediction.

### 3.3. Optimization Strategy

#### 3.3.1. RBE Preprocessing

Certain inducing factors will increase the RBE numbers of the partial page in the block. In addition, the arithmetic average can weaken the endurance difference caused by the increase of the RBE numbers of the partial page, reflecting the overall level of endurance of the flash memory block. The erase operation acts on all pages in the flash memory block. If the RBE numbers of a page exceeds the upper limit of ECC error correction, the block will be marked as a bad block. Therefore, the maximum value of page RBE is an effective endurance level predictive input feature.

The standard deviation of the RBE numbers between pages can effectively reflect the difference in endurance between pages. The difference in endurance between pages increases as the endurance decreases, which means that the standard deviation of the RBE numbers can reflect changes in endurance. At the same time, when some non-local disturbances cause overall changes in the RBE numbers of pages within a block, the arithmetic mean will be greatly affected. However, the standard deviation describing the degree of difference can well shield these integrity negative effects of disturbances.

**Figure 3:**
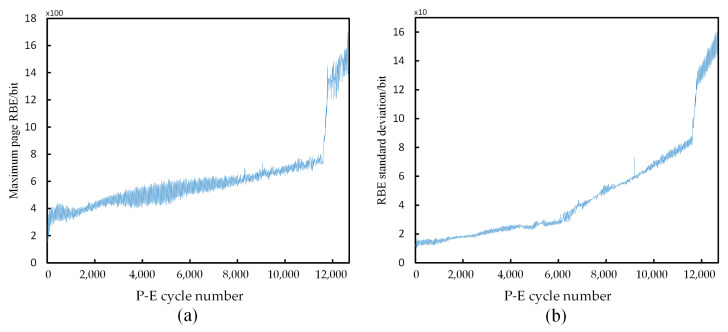
(**a**) The relationship between the maximum RBE numbers of flash memory page and P-E cycle number; (**b**) The relationship between page standard deviation and P-E cycle number.

Figure 3 shows a statistical graph of the maximum value of the RBE numbers and standard deviation of a certain block of pages as the number of P-E cycles increases. After ignoring the jitter, the figure shows a monotonous upward trend with the number of P-E cycles, which provides significant rules for machine learning algorithms to learn.

#### 3.3.2. Transient Error

The transient error caused by the RTN phenomenon has uncertainty: the uncertain drain current causes the threshold voltage to shift randomly, resulting in an uncertain change in the page RBE numbers. Therefore, the page RBE numbers obtained by the test jitters violently as the number of P-E cycles $N_{c}$ increases, causing significant noise. Since the page RBE numbers is small at the initial stage of wear, and the amount of page RBE change caused by the increase in $N_{c}$ is not significant, this kind of jitter noise has a great negative impact on endurance level prediction accuracy.

Repeating the P-R-E cycle for a predefined duration and taking the average value of the page RBE can effectively reduce the page RBE numbers noise caused by the RTN phenomenon. However, continuous read operation cannot be used instead of continuous P-R-E operation. Performing a continuous read operation after a single programming operation can ensure that the page RBE numbers obtained each time is under the same $N_{c}$. However, in multiple consecutive reading operations, the reading disturbance makes each reading operation affect the result of subsequent reading, and the multiple reading operations are not independent. After the erase operation, the memory cell is approximately restored to the same state, so each P-R-E cycle can be considered independent and does not affect each other. In addition, the RTN phenomenon mainly occurs during the programming operation and for a period of time after it. The RTN phenomenon does not significantly disturb the threshold voltage during multiple continuous read operations. Therefore, the continuous read operation after programming does not improve the negative impact of the RTN phenomenon. Because the maximum value operation is a nonlinear transformation, the sequence of page RBE numbers preprocessing and transient error optimization strategy will have a potential impact on prediction accuracy.

## 4. Experiments and Analysis

### 4.1. Test Platform

#### 4.1.1. Test Platform Architecture

**Figure 4:**
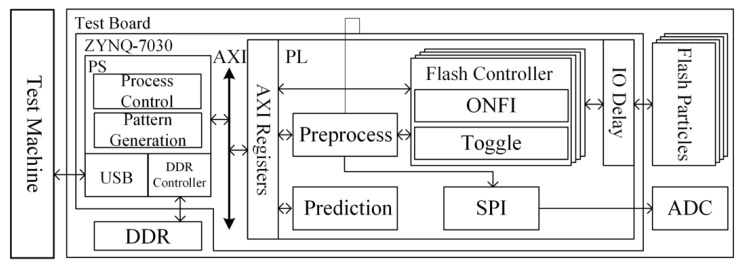
Overview of the Flash test platform.

A scheme is realized by the Xilinx ZYNQ-7000 series xc7z030ffg676-2 SoC chip (hereafter referred to as ZYNQ-7030) to build a NAND Flash test platform. The test platform consists of a host computer and multiple test boards, as shown in Figure 4. A graphical user interface (GUI) test program runs in the host computer, and multiple test boards are controlled by USB transmission. There are eight test sockets on the test board, and eight BGA132/152 packaged flash memory particles can be tested in parallel at the same time.

The test machine is responsible for flash memory specification setting, test process setting, and data storage. Each test board has a ZYNQ-7030 chip, which exchanges data with eight test flash memory particles through GPIO. ZYNQ-7030 can be divided into Processing System (PS) running firmware and Programming Logic (PL) based on Kintex-7 FPGA. The core of the PS is dual-core Cortex-A9, which is mainly responsible for flash memory particle initialization, test flow control, programming pattern drawing, etc. The firmware will automatically generate a test process loop according to the test process parameters transmitted by the host computer, and control the PL-side Flash interface protocol controller module to complete the corresponding command operations through the AXI bus register.

The PL is mainly responsible for functions such as flash interface control, test data processing, and endurance class prediction, including Flash interface protocol controller module, input acquisition and preprocessing module, machine learning prediction algorithm module, SPI protocol control module, etc. There are eight independent Flash interface protocol controller modules, each of which controls the flash memory particles of the channel. The controller module interacts with the PS firmware through the AXI bus register, and the information obtained during the test will pass through the input acquisition and preprocessing module, and then be transmitted to the PS. The SPI protocol control module controls the external 16 bit resolution ADC chip, which can obtain the current value of the flash memory particles at any time and calculate the instantaneous power consumption based on this.

#### 4.1.2. Test Platform Cost

The test platform can test 64 × 8 flash memory particles at the same time. For a single flash memory particle with a block size of 1024, 50,000 P-E cycles only need 1213 min, and 5 repeated PRE cycles for 1000 blocks only need 261 min. The former corresponds to the data acquisition of the model building process, and the latter corresponds to the data acquisition of the actual application process.

In terms of predictive circuit modules, the use of PL-side FPGA can greatly shorten the time-consuming prediction of endurance levels. After testing, a single prediction of the prediction module under a 100 Mhz clock requires only about 37 us, while a single prediction implemented by PS-side embedded programming requires 108 us.

In terms of resource consumption, the PL-side FPGA hardware resource occupancy is shown in Table 1. The test platform and its proportion are calculated in the case of a 32-bit wide arithmetic unit. In order to achieve highly parallel testing, a single ZYNQ-7030 chip has eight channels internally instantiated, and each channel is divided into four parallel modules to realize multi-CE embedded testing. Each way, the parallel module supports three kinds of interface protocols. In the prediction circuit module, the CORDIC calculation module occupies a higher number of LUTs and Registers resources, which occupy 3925 and 3904, respectively. When the bit width of the calculation unit is reduced to 16 bit, the related resource consumption of the CORDIC calculation module drops significantly to 1159 and 1148.

### 4.2. Experiment Methodology

#### 4.2.1. Experiment Method

The flash memory particles selected in the experiment are the same batch of MT29F256G08EBHAFJ4 (NW911) chips from Micron. The flash memory particle type of this model is 3D TLC, whose block size is 2304, and the page capacity is 18,588 bytes. In the experiment, the number of consecutive P-R-E cycles $T_{\varepsilon }$ = 5, $T_{\alpha }+T_{\varepsilon }=50$, a set of sample data can be obtained every 50 times of programming, and a total of 96 flash memory blocks are tested for each endurance stage. The programming pattern adopts a PS/PL mixed pseudo-random pattern.

The experiment uses a multi-classification model. There are four labels in the output result of this experiment, which are the maximum level of RBE on the page after 100, 200, and 500 P-E cycles and the level of the number of remaining P-E cycles, which are marked as labels 1-4 in order. As shown in Table 2, each label contains four categories:

- (a) When the number of P-E cycles is used as the dimension, the level of the remaining P-E cycles is used as the output. The category boundaries between different levels are 500, 2500, and 4500 remaining cycles. The four levels of the remaining P-E cycles are [0,500),[500,2500),[2500,4500),[4500,∞), recorded as level 1–4.
- (b) When RBE is the dimension, the level of the maximum page RBE when the number of P-E cycles $N_{c}=N_{c}^{\prime }+N_{i}$ is output as the sample point when $N_{c}=N_{c}^{\prime }$, and the category boundaries are 400, 700, and 1000 bits. The four levels of the maximum value of the page RBE are [0,400),[400,700),[700,1000),[1000,∞), marked as level 1–4. The values of $N_{i}$ are 100, 200, 500, and there are three tags depending on the difference of $N_{i}$.

The experiment uses SVM algorithm for model training by default, and the verification method uses five-fold cross-validation. We conducted four independent model trainings. Each training is based on the training data set of one label, and the training data of the remaining three labels is discarded. Since the total number of P-E cycles of the sample flash memory block is wide, and the maximum RBE value at the initial stage of life is generally higher than 300, it is very close to the category boundary 400 of category 1 and category 2. This phenomenon leads to an imbalance in the number of samples in different categories for each label, and the imbalance in the number of samples in different categories is not consistent on different labels. Therefore, before four independent model trainings, we balance the number of samples of different categories for each label, and reduce the number of samples of the three larger categories to the smallest number of category samples by random selection—the total number of samples for label 1 was found to be 6576, label 2 was 8272, label 3 was 8952, and label 4 was 15,208.

#### 4.2.2. Evaluation Indicators

1. Confusion Matrix

The confusion matrix is a visual numerical matrix used to reflect the classification results of a supervised machine learning model. Various indicators of the classifier model are calculated based on the confusion matrix. The confusion matrix of the L classifier model is a square matrix of L×L, which can intuitively reflect the distribution of each actual category and output category. Each row of the confusion matrix belongs to the same actual category, and each column belongs to the same output category.

2. Numerical Indicators

The two-class model commonly uses Accuracy (A), Precision (P), Recall (R), and F1-score ($F_{1}$) to measure the pros and cons of the model. However, in the multi-class model, the increase in the number of rows and columns of the confusion matrix leads to ambiguous indicator definitions, so corresponding changes are needed. The expression and meaning of the numerical indicators of the classification model are shown in Table 3. Among them, the accuracy and recall rate in the multi-classification model are divided into three categories: macro, micro, and weighted, and the Kappa coefficient is introduced.

3. ROC Curve

The ROC curve is often used for model comparison and threshold screening in the case of classification. The AUC value of the area under the curve can intuitively reflect the pros and cons of performance. The larger the AUC value, the better the performance. In a multi-class model, each category corresponds to a ROC curve, and it is necessary to ensure that the categories are the same when comparing models.

In summary, when comparing the prediction results, we will compare the accuracy rate *A*, macro accuracy *macro−P*, macro recall rate *macro−R*, *macro-F*1 score, Kappa coefficient *K*, and roc curve.

### 4.3. Analysis

#### 4.3.1. Comparison of the Results of Different Labels

This experiment uses Binary Relevance technology to transform the multi-label multi-classification model into L single-label multi-classification models. Each label is trained separately to obtain the prediction result, and the results of different labels are compared. The numerical indicator results are shown in Table 4.

The numerical indicators of label 1 are the best. The first four indicators all reach 95.8%, the Kappa coefficient is about 94.4%, and the K value greater than 90% means that the model has extremely high consistency. The effect of the numerical indicators of label 2 is followed closely. The first four indicators are about 95.2–95.3%; the gap is not big. The prediction effect of the model of label 4 is the worst. The first four indicators are about 89.7%, and the K value is only 86.4%.

**Figure 5:**
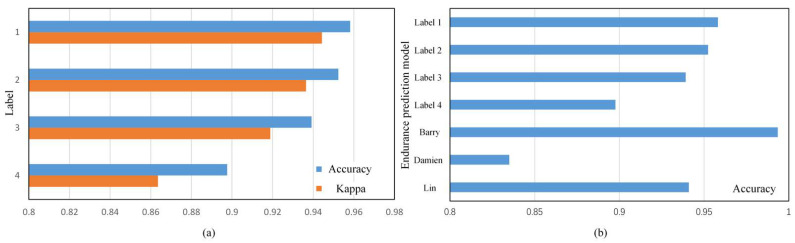
(**a**) Comparison of model accuracy and Kappa coefficient of different labels; (**b**) Comparison of the accuracy of models in other studies.

Figure 5a visually compares the correct rate A and Kappa coefficient K of the models of different labels through the bar graph. The difference between labels 1 to 3 is not big, label 1 is the best, and label 4 is obviously different. The indicator gap between the four labels is related to the classification basis. Labels 1 to 3 are divided into categories based on the RBE numbers, which essentially predicts the changes of certain parameters after a certain number of times in the future. At the same time, the difference between tags 1 to 3 is that the value of $N_{i}$ is different. In addition, the value of $N_{i}$ reflects the number of times the predicted target is away from the current state. Tag 1 has the smallest gap and tag 3 has the largest. The smaller the gap means the smaller the change based on the current state, and the higher the accuracy of the prediction will naturally be. Tag 4 is divided into categories based on the number of P-E cycles. Essentially, it judges the current endurance stage based on the characterization of the current endurance parameters, and it also needs to determine the total endurance range. The large difference in endurance and mischaracterization between the flash memory particles greatly weakens the model’s ability to judge.

The comparison between the models corresponding to the four labels and the endurance prediction models of other researchers is shown in Figure 5b. Barry’s scheme worked best, achieving a 99.4% correct rate. However, the number of negative samples in the study only accounted for 0.03%, which greatly reduced its reliability. The correct rates of labels 1–3 and Lin’s models in this scheme are about 94–96%, followed by label 4. These models are ahead of the 83.5% correct rate of Damien’s scheme. Excluding the unreliable Barry scheme due to extremely unbalanced samples, the model accuracy rate of this scheme is in the first echelon in this field. Compared with the two-class judgment of other schemes, this scheme adopts a multi-classification model, and the increase in the number of categories makes it more abundant in application scenarios. In addition to basic bad block warning, the model of this solution can also be used for wear leveling strategies, factory screening and rating, etc.

**Figure 6:**
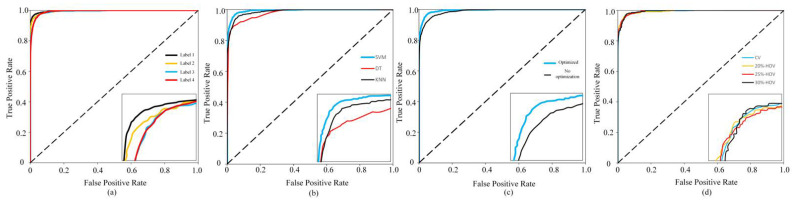
(**a**) ROC curves of models with different labels; (**b**) ROC curves of different algorithms; (**c**) ROC curves of models with or without transient error optimization; (**d**) ROC curves of models with different verification method.

Since the AUC value of category 4 of each model is about 0.99, the upper left corner area is enlarged to the lower right corner for display. As shown in Figure 6a, the AUC value relationship of tags 1–3 is consistent with the correct rate relationship, that is, $AUC_{1}>AUC_{2}>AUC_{3}$.

The ROC curve of category 4 of labels 1 to 3 reflects the model’s prediction of bad blocks, because the boundary of category 4 is close to the critical value of bad block judgment. This shows that the pros and cons of the bad blocks predicted by tags 1–3 are also consistent with the overall pros and cons of the model. However, the special case is that the ROC curves of label 4 and label 3 are very close, but there is a gap between the two in numerical indicators. There are two main reasons for this phenomenon: first, the classification dimensions of label 3 and label 4 are not consistent, and the meaning of category 4 is not the same. It is meaningless to directly compare the ROC curves of the two categories 4; second, the selected numerical indicators. It reflects the overall situation of the four categories, and there are differences between the local and the whole. In fact, the correct rate A of each category is calculated separately, and the correct rates A of label 3 and label 4 to category 4 are 96.27% and 96.26%, respectively, which are very close. However, the correct rate A of label 3 for categories 2 and 3 is 97.64% and 95.26%, while the correct rate A of label 4 for categories 2 and 3 is only 93.55% and 92.18%, which is a large gap.

Considering the evaluation indicators and actual application scenarios, this paper believes that the prediction model of label 3 is the best because the numerical indicator of label 4 is low. When the difference between the internal numerical indicators of labels 1 to 3 is not large, the value of $N_{i}$ of label 3 is larger, which means that label 3 can make early warning and decision-making in actual application scenarios. Therefore, when comparing other variables in the follow-up, they will all be discussed in the case of label 3.

#### 4.3.2. Comparison of Results of Different Algorithms

**Figure 7:**
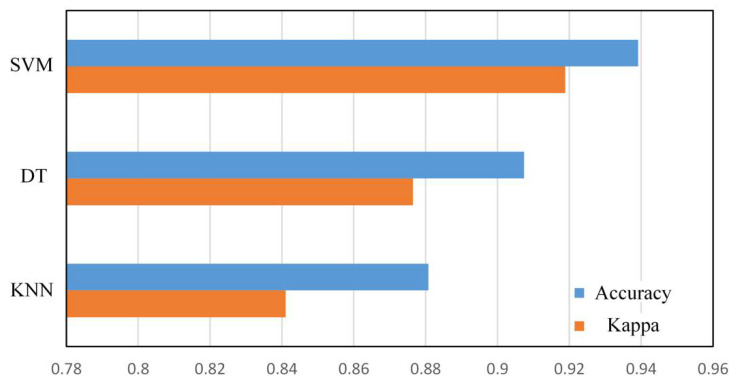
Comparison of model accuracy and Kappa coefficient of different algorithms.

In addition to the default SVM algorithm, we also use the DT algorithm and the KNN algorithm to perform model training on the same training set. The results are shown in the Table 5. Figure 7 shows Accuracy and Kappa coefficient of different algorithms. The SVM algorithm has achieved the best results in all the numerical indicators in the table, which is about 3% to 4% higher than the DT algorithm, and about 5% to 8% higher than the KNN algorithm. At the same time, this experiment has conducted sample balance processing between each category, considering the KNN algorithm’s classification disadvantages of unbalanced sample data sets; in actual situations, when the endurance level prediction scheme is applied, the KNN algorithm may be more disadvantageous in accuracy.

It can be seen from the ROC curve in Figure 6b that the SVM algorithm is still the best in performance of the AUC value, while the DT algorithm is the worst, and the gap is obvious. Because the ROC curve for category 4 reflects the model’s classification performance in the critical value of bad block judgment, and is an important indicator of the pros and cons of the bad block early warning function, the DT algorithm has a great disadvantage in this important function.

#### 4.3.3. Analysis of Transient Error Optimization Effect

1. Comparison of Optimized and No Optimization

Comparing the effect of optimization with or without transient errors will inevitably lead to an imbalance in the number of category samples in one of the cases. Therefore, the weighted-P indicator is added to the statistical table of numerical indicators of the model with or without optimization, as shown in Table 6.

The input of the non-optimized model takes the last time of the $T_{\alpha }$ cycle, and the input of the optimized model uses the first average processing method. From a comparison of Table 6, it can be seen that there is a huge gap in the numerical indicators of the model with or without transient error optimization. The accuracy rate A of the optimized model is 7.9% higher than that of the non-optimized model. Among the other five indicators, the optimized model is about 7% to 10% higher than the non-optimized model, which is a significant improvement. This is because the transient error optimization strategy can significantly reduce the jitter noise in the endurance data, so that the machine learning algorithm can better analyze the intrinsic relationship between the endurance level and the input vector.

The ROC curve in Figure 6c shows that the AUC value of the optimized model is higher than that of the non-optimized model, so the optimized model judges bad blocks more accurately. The comparison result fully illustrates the necessity and correctness of the transient error optimization strategy.

2. Comparison of Optimization Order

The sequence problem of the maximum/standard deviation operation and the transient error optimization operation will cause the difference of the input vector after the transient error optimization, which is essentially caused by the characteristics of the nonlinear transformation. The previous forecasting models are all optimized before processing. Table 7 shows the numerical indicator results of earlier transient optimization and later transient optimization.

It can be seen from the table that pre-optimization is better in the prediction results. The accuracy rate A has achieved a lead of 2.4%, and the other indicators have achieved a lead of 2% to 3%. The result is related to the theoretical basis of the optimization strategy. The transient error optimization strategy is based on the theoretical situation that probability $p=\psi \left(N_{c}\right)$ can be considered as a fixed value, when $N_{c}$ is approximately constant. However, the function $\varphi \left(N_{c}\right)$ of different storage units is different, owing to which the theoretical situation applies only to the same storage unit or page. Post-optimization will cause the optimization strategy to deviate from the theoretical situation. At the same time, in the early stage of endurance when the page RBE numbers change little, the gap between the RBE numbers pages is little. The errors caused by various disturbance factors account for a relatively large amount. The post-optimization will greatly weaken the effect of transient error optimization. The pre-optimization can ensure that the input vector of $f\left(S_{k}\right)$ comes from the same flash page, so that the theoretical situation is applicable and no negative effects will occur. Therefore, the pre-optimization method can achieve better optimization results.

#### 4.3.4. Analysis of Validation Method and Feature Correlation

1. Comparison of Different Validation Methods

The performance of classification model results is evaluated and compared in a five-fold cross-validation method. The advantage of this method is to reduce the statistical uncertainty of the average test error estimation, so as to facilitate model comparison and result analysis. In order to avoid misjudgments caused by differences in validation methods, we also compared the prediction results of the five-fold cross-validation and different ratios of Hold-Out validation methods. The Hold-Out ratios are selected as 20%, 25%, and 30%.

According to the comparison results of the indicators in Table 8, the best indicator is the Hold-Out method with a ratio of 20%, and the worst is the Hold-Out method with a ratio of 30%. The accuracy difference between the two is about 1.7%. The Kappa coefficient gap is about 2.3%. In fact, the prediction result of the Hold-Out method changes greatly due to the difference in the selection of the test set. Taking the 20% ratio Hold-Out method as an example, the accuracy rates A of the five repetitive training models with the same data set are 94.52%, 92.99%, 93.83%, 93.60%, and 94.66%, and the difference between the maximum and minimum values is about 1.67%. Taking into account the indicator fluctuations caused by the difference in the selection of the test set, it can be considered that the numerical indicators of the models in the four cases are very close.

The ROC curve in Figure 6d also confirms this conclusion. The ROC curves of the four models are very close. Therefore, the Hold-Out method with a separate test set can still obtain almost the same evaluation index, indicating that the model obtained by the endurance level prediction scheme can still achieve excellent prediction results in the additional test set.

2. Feature Correlation

At present, the features of the experiment are the arithmetic mean, maximum, and standard deviation of the page RBE numbers, as well as the number of P-E cycles and the duration of erasure. When performing feature analysis, the Pearson correlation coefficient r can be used to measure the linear correlation between the various dimensions of the input vector. When its value is close to 1, it means that the redundancy of the input vector space is large, and the dimension of the input vector can be simplified. Through calculation, the Pearson correlation coefficient $r\left(RBE_{a},RBE_{s}\right)$ between the arithmetic mean of the page RBE numbers and the standard deviation is:

(1) $$r\left(RBE_{a},RBE_{s}\right)=\frac{\sum_{i}\left(X_{i}-\bar{X}\right)\left(Y_{i}-\bar{Y}\right)}{\sqrt{\sum_{i}{\left(X_{i}-\bar{X}\right)}^{2}}\sqrt{\sum_{i}{\left(Y_{i}-\bar{Y}\right)}^{2}}}=0.9790$$

The value is extremely close to 1, which means that there is a strong linear correlation between the two vectors. When the two are used as the model input vector dimensions at the same time, the recognition of the feature relationship between the input and the output is of minimal help. At the same time, the prediction circuit needs to perform parallel calculations on various dimensions of input. If the input dimensions can be reduced, the hardware resource consumption will be greatly reduced.

As shown in Table 9, the PCA dimensionality reduction method reduces the input from five dimensions to four. Through comparison, it can be found that the complete input still achieves the best prediction effect, but the lead is extremely small. Excluding the two cases of RBE arithmetic mean/standard deviation, the difference is too small to be ignored. Considering that the arithmetic mean can shield the local disturbance, and the standard deviation can shield the overall disturbance, the linear correlation between the two vectors is extremely strong, indicating that the impact of the endurance change on the overall disturbance and the local disturbance is positively correlated.

**Figure 8:**
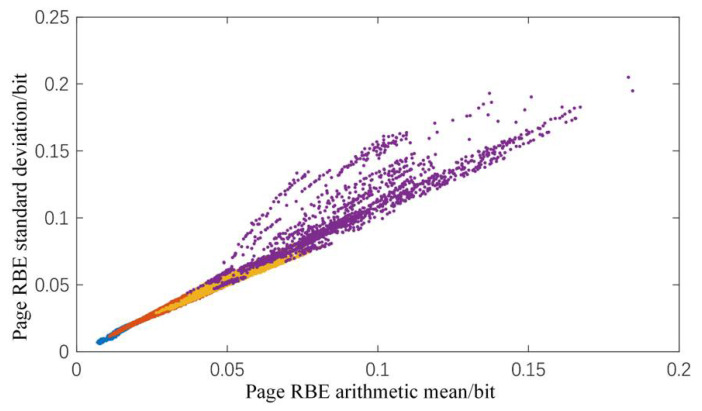
The distribution of the arithmetic mean and standard deviation of the sample points of the data set.

Figure 8 shows that the arithmetic mean and standard deviation are roughly linear distributions, which are consistent with the results. The model indicator using the PCA dimensionality reduction method is very close to the complete input model indicator, but this method requires dimensionality reduction through a certain function transformation, which will add additional hardware resources. The method of removing the arithmetic mean/standard deviation of the page RBE reduces the consumption of hardware resources on the premise that the difference between the arithmetic mean/standard deviation and the complete input is negligible. Therefore, the input of the standard deviation will be removed in the implementation of the specific scheme.

### 4.4. Application

The endurance level prediction model has many practical application scenarios. The paper designs a simple warning strategy for bad blocks based on the prediction scheme introduced. In the actual application scenario of bad block warning, the prediction model will face the problem of recall rate. Assuming that a block will become a bad block after a certain number of programming times, the recall rate determines the probability that the prediction model can be used to successfully judge and give an early warning. The recall rate is the most important evaluation indicator in the early warning of bad blocks. In data-sensitive fields, users stop using the flash memory when the usage of flash memory reaches half of the nominal value because the method can make the recall rate reach 100%. Even if the method will cause the real usage rate of flash memory to be much lower than 10%, it is necessary to ensure that no bad blocks are missed. Therefore, the prediction model applied to the bad block early warning strategy needs to achieve the following goals:

(1) Improve the recall rate of the bad block warning strategy as much as possible.
(2) On the premise of ensuring goal 1, try to increase the real utilization rate of flash memory, that is, postpone the bad block warning time.
(3) Reduce the wake-up frequency of the prediction program and prediction circuit to reduce the burden on the SSD controller.

**Figure 9:**
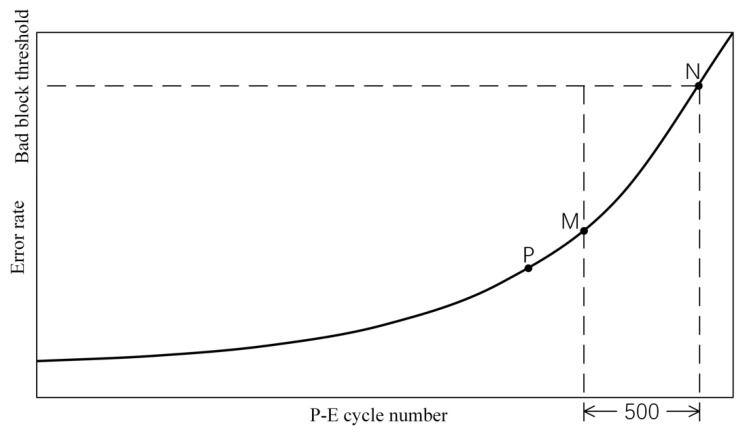
Schematic diagram of the flash block error rate and the number of P-E cycles.

Based on the above objectives, this paper designs a comprehensive strategy for early warning of bad blocks. Take Figure 9 as an example—the curve in the figure represents a schematic curve of the error rate of the flash memory block as a function of the number of P-E cycles. Assuming that the flash memory reaches the critical value of bad blocks at point N, an uncorrectable data error occurs. The number of P-E cycles between point M and point N differs by 500. Point P is the first time that the predictive model judges the block to be a positive type (assuming that Bad block) moment. Before point P, the bad block warning strategy wakes up the endurance level prediction circuit during every A programming operation. After the P point, it is changed to wake up once every B programming operation, B < A. In this way, the prediction circuit can be called with a lower wake-up frequency during the endurance stage with lower risk, and frequent predictions when approaching the end of the endurance period, in order to take into account resource consumption and the accuracy of the early warning strategy. In the judgment of bad blocks, the category 4 of the prediction model labels 1 to 3 or category 1 of the label 4 are regarded as the positive type, because they both mean that the prediction result of the flash memory block is located at point M and to the right. The prediction circuit is frequently awakened after point P. If C consecutive prediction results are positive, an early warning is sent to the controller.

Let A be 200 and B be 50, and use the prediction model of label 3 to test. After testing 96 sample blocks, the program successfully provided early warning for 93 blocks, with a success rate of about 96.9%. The recall rate of the model category 4 is only 89.90%, which shows that the bad block early warning strategy can successfully improve the accuracy of the endurance class prediction model in practical applications under the condition of low wake-up rate.

## 5. Conclusions

In order to effectively prolong the service life of flash memory and avoid the loss caused by sudden failure, this paper conducts related research on flash memory endurance, proposes a flash memory endurance grade prediction scheme based on the SVM algorithm, and designs a high parallel test platform and low time-consuming endurance prediction module based on FPGA. We research and analyze the feature quantities closely related to the endurance changes in the flash memory, and determine that the model takes the block as the object. The page RBE numbers, the number of P-E cycles, and the erase duration in the block are used as the input feature quantity, and the output is the remaining lifetime level, or RBE numbers level after 100/200/500 P-E cycles. This scheme adopts a variety of strategies to reduce the negative interference in the forecasting process in a targeted manner. The prediction module is realized based on the ZYNQ-7030 chip. The SVM decision model is deconstructed and the parallel multiplication structure is designed to realize the highly multiplexed pipelined calculation. The prediction module only needs 37 us per time, which greatly reduces the time consumption of prediction.

The method uses multi-category evaluation indicators to analyze five aspects: four tags achieved 89.77–95.82% accuracy, each evaluation indicator is in the leading echelon, and the increase in the number of categories expands the scope of application. Compared with DT and KNN, the SVM model of the RBF kernel function achieved a lead of 3–8%. The model using the transient error optimization strategy achieved an indicator increase of 7–10%, and pre-optimization leads up to 2% to 3%. Cross-validation and Hold-Out validation results show that the model can still achieve the same prediction effect in the additional test set. Pearson correlation coefficient analysis shows that the impact of the endurance change on the overall disturbance and the local disturbance is positively correlated. Finally, the bad block early warning strategy designed based on the proposed model can successfully achieve early warning for 96.9% of the blocks.

## Figures and Tables

**Table 1:** PL Hardware Resource Cost.

Resource	Test Plat-Form (32 bit)	Prediction Circuit		Ratio (32 bit)
		32 bit	16 bit	
Slice LUTs	34,199	5947	1701	44%
Slice Registers	33,732	7689	1941	21%
RAMB36E1	142	28	14	54%
RAMB18E1	9	0	0	2%
DSP48E1s	20	20	8	5%

**Table 2:** The labels and categories.

Labels	Category 1	Category 2	Category 3	Category 4
1 (P-E cycles)	[0,500)	[500,2500)	[2500,4500)	[4500,∞)
2 (RBE when $N_{i}=100$)	[0,400)	[400,700)	[700,1000)	[1000,∞)
3 (RBE when $N_{i}=200$)	[0,400)	[400,700)	[700,1000)	[1000,∞)
4 (RBE when $N_{i}=500$)	[0,400)	[400,700)	[700,1000)	[1000,∞)

**Table 3:** The expression and meaning of the numerical indicators of the classification model.

Indicators	Two-Class	Multi-Class	Meaning
Accuracy	$\frac{TP+TN}{TP+TN+FP+FN}$	$\frac{1}{S}\cdot \sum_{m}A_{m,m}$	Overall prediction accuracy rate
Precision	$\frac{TP}{TP+FP}$	$\begin{array}{c} macro-P=\frac{1}{L}\sum_{n}\frac{A_{n,n}}{\sum_{m}A_{m,n}}\\ micro-P=Accuracy\\ weighted-P=\sum_{n}\frac{A_{n,n}\cdot \sum_{m}A_{n,m}}{S\cdot \sum_{m}A_{m,n}} \end{array}$	Ratio of correct predicted value
Recall	$\frac{TP}{TP+FN}$	$\begin{array}{c} macro-R=\frac{1}{L}\sum_{m}\frac{A_{m,m}}{\sum_{n}A_{m,n}}\\ micro-R=weighted-R=A \end{array}$	Ratio of correct true value
F1-score	$\frac{2P\cdot R}{P+R}$	$\frac{2P\cdot R}{P+R}$	Precision/recall rate trade-off value
Kappa		$\frac{S\cdot \sum_{m}A_{m,m}-\sum_{n}\left(\sum_{m}A_{m,n}\right)\left(\sum_{m}A_{n,m}\right)}{S^{2}-\sum_{n}\left(\sum_{m}A_{m,n}\right)\left(\sum_{m}A_{n,m}\right)}$	Biased consistency indicators

**Table 4:** Statistical table of model numerical indicators of different labels.

Labels	A	macro−P	macro−R	$macro-F_{1}$	K
1	0.958222	0.958532	0.958222	0.958377	0.944296
2	0.952369	0.953216	0.952369	0.952793	0.936493
3	0.939173	0.940106	0.939173	0.939639	0.918897
4	0.897679	0.897441	0.897679	0.897561	0.863572

**Table 5:** Statistical table of model numerical indicators of different algorithms.

Labels	A	macro−P	macro−R	$macro-F_{1}$	K
SVM	0.939173	0.940106	0.939173	0.939639	0.918897
DT	0.907391	0.909162	0.907391	0.908275	0.876521
KNN	0.880779	0.889564	0.880779	0.885149	0.841038

**Table 6:** Statistical table of numerical indicators of optimization and no optimization.

Labels	A	macro−P	macro−R	$macro-F_{1}$	K
Optimized	0.939173	0.940106	0.939173	0.939639	0.940106
No optimization	0.860097	0.8575992	0.858039	0.857819	0.864401

**Table 7:** Statistical table of numerical indicators of optimization order.

Labels	A	macro−P	macro−R	$macro-F_{1}$	K
Pre-optimization	0.939173	0.940106	0.939173	0.939639	0.940106
Post-optimization	0.915602	0.915280	0.917297	0.916288	0.916810

**Table 8:** Statistical table of numerical indicators of different validation methods.

Labels	A	macro−P	macro−R	$macro-F_{1}$	K
Five-fold cross-validation	0.939173	0.940106	0.939173	0.939639	0.918897
20% Hold-Out	0.945247	0.945659601	0.94525632	0.945457917	0.92699593
25% Hold-Out	0.934307	0.936912156	0.934306569	0.935607549	0.912408759
30% Hold-Out	0.927992	0.929085804	0.927991886	0.928538523	0.903989182

**Table 9:** Statistical table of numerical indicators of input dimension reduction.

Labels	A	macro−P	macro−R	$macro-F_{1}$	K
Five inputs	0.939173	0.940106	0.939173	0.939639	0.918897
No arithmetic mean	0.934002	0.935897511	0.934002433	0.934949012	0.912003244
No standard deviation	0.933394	0.935000947	0.933394161	0.934196863	0.911192214
PCA method	0.937196	0.939040182	0.937195864	0.938117116	0.916261152
